# Assessing the fragility index of randomized controlled trials supporting perioperative care guidelines: A methodological survey protocol

**DOI:** 10.1371/journal.pone.0310092

**Published:** 2024-09-12

**Authors:** Margarita Otalora-Esteban, Martha Beatriz Delgado-Ramirez, Fabian Gil, Lehana Thabane

**Affiliations:** 1 Department of Anesthesiology and Pain Medicine, Toronto General Hospital, University Health Network, University of Toronto, Toronto, ON, Canada; 2 Department of Clinical Epidemiology and Biostatistics, School of Medicine, Pontificia Universidad Javeriana, Bogotá D.C, Colombia; 3 Department of Anesthesiology, San Ignacio University Hospital, School of Medicine, Pontificia Universidad Javeriana, Bogotá D.C, Colombia; 4 Department of Health Research Methods, Evidence and Impact, McMaster University, Hamilton, ON, Canada; 5 Biostatistics Unit, St Joseph’s Healthcare Hamilton, Hamilton, ON, Canada; 6 Faculty of Health Sciences, University of Johannesburg, Johannesburg, South Africa; IRCCS: IRCCS Ospedale San Raffaele, ITALY

## Abstract

**Introduction:**

The Fragility Index (FI) and the FI family are statistical tools that measure the robustness of randomized controlled trials (RCT) by examining how many patients would need a different outcome to change the statistical significance of the main results of a trial. These tools have recently gained popularity in assessing the robustness or fragility of clinical trials in many clinical areas and analyzing the strength of the trial outcomes underpinning guideline recommendations. However, it has not been applied to perioperative care Clinical Practice Guidelines (CPG).

**Objectives:**

This study aims to survey clinical practice guidelines in anesthesiology to determine the Fragility Index of RCTs supporting the recommendations, and to explore trial characteristics associated with fragility.

**Methods and analysis:**

A methodological survey will be conducted using the targeted population of RCT referenced in the recommendations of the CPG of the North American and European societies from 2012 to 2022. FI will be assessed for statistically significant and non-significant trial results. A Poisson regression analysis will be used to explore factors associated with fragility.

**Discussion:**

This methodological survey aims to estimate the Fragility Index of RCTs supporting perioperative care guidelines published by North American and European societies of anesthesiology between 2012 and 2022. The results of this study will inform the methodological quality of RCTs included in perioperative care guidelines and identify areas for improvement.

## 1. Introduction

### Background

The use of statistically significant findings in scientific literature has come under scrutiny in recent years, as has the misuse of statistical tests and misinterpreting results [1, 2]. A significant and seemingly high-quality portion of evidence-based medicine consists of randomized trials. Limitations of Randomized Clinical Trials (RCTs) include incorrect statistical inference, low internal or external validity, misinterpretation of statistical approaches, publication bias, and difficulty applying findings to individual patients [3]. Conclusions taken from trial findings may be questioned due to their fragility, particularly when little adjustments have a significant impact on the result [4]. The fragility index (FI) measures the minimum number of conversions from nonevent to event in a treatment group to shift the P-value over the 0.05 threshold [4]. The FI family of indices includes the FI, the Reverse Fragility Index (rFI), the Fragility Quotient (FQ), Incidence fragility index (FIq), and the Generalized fragility index (GFIq) [5]. These measures can provide valuable information on the statistical stability of trial results and the susceptibility to misinterpretation [6–10].

The limitations of conducting RCTs in perioperative medicine, including frequently non-statistically significant results and susceptibility to spin bias, have prompted more comprehensive research methods to facilitate comparison between studies [11]. The use of fragility assessment as a complementary measure to determine the stability of study results has been proposed as a potential solution [12, 13]. Its use has been extended to evaluate the robustness of RCT results in Clinical Practice Guidelines (CPG) [6, 14, 15].

Previous studies evaluating fragility in anesthesiology RCTs have produced similar results to those reported in other clinical areas, with a median FI of 3 (IQR,1–7) [11, 16–19].

A recent assessment of the evidence supporting the North American Society of Anesthesiology and the European Society of Anesthesiology CPG found that less than one-fifth of the recommendations are supported by Grade A evidence [20].

Spin in abstracts of RCTs published in high-impact anesthesia journals has been reported between 40–54%, misrepresenting validity and potentially impacting clinical decisions [16, 21].

These findings underscore the importance of Meta-research-related initiatives’ importance in identifying methodological strengths and weaknesses and promoting evidence-based science by eliminating ineffective research practices [22, 23].

### Objectives

#### a. Primary objective

To assess the fragility of RCTs with dichotomous outcomes supporting perioperative care guidelines of the North American and European societies of anesthesiology between 2012 and 2022.

#### b. Secondary objective

To explore randomized controlled trials attributes influencing statistical fragility

## 2. Methods and analysis

### Study design

This study corresponds to a methodological survey of RCTs supporting perioperative care guidelines published by North American and European societies of anesthesiology between 2012 and 2022.

This protocol is registered in OSF registries (Registration DOI: https://doi.org/10.17605/OSF.IO/8KBPE).

### Eligibility criteria

CPGs will be selected based on the following eligibility criteria:

1. Inclusion criteria:
  - Perioperative evidence-based guidelines for perioperative care medicine interventions aimed at anesthesiologists.
  - English-written guidelines from the North American societies (the United States, Canada, and the United Kingdom) and European societies.
  - Released between 2012 and 2022.
  - Must include an explicit statement identifying it as a "guideline."
2. Exclusion criteria:
  - Critical care and chronic pain medicine perioperative evidence-based guidelines.
  - Practice recommendations, practice advisories, or consensus statements.
  - Older versions of the same guidelines, based on the year of publication.

Selected CPGs will be reviewed to identify all possible RCTs supporting the recommendations within each guideline; without language limitations. Each of the trials will be screened for eligibility using the following criteria:

1. Inclusion criteria:
  - Human clinical trials with two arms using a 1:1 allocation ratio
  - The trial uses a binary outcome.
  - Intervention studies in the adult, obstetric, and pediatric populations.
2. Exclusion criteria:
  - Not using a two-parallel-arm trial design or a two-by-two factorial RCT.
  - Non-inferiority trials

### Search strategy

Before conducting the comprehensive search, a preliminary search was made to ensure study viability and to identify the list of eligible anesthesia societies “S1 Appendix”. The search strategy was developed with the assistance of a medical librarian.

We will conduct a two-step comprehensive search strategy. In the first step, we will search for CPGs published by the North American and European Societies of Anesthesiology between 2012 and 2022. In the second step, we will identify RCTs supporting the recommendations in these guidelines.

The search will be conducted in MEDLINE, Embase, TRIP Database, and the North American and European Societies of Anesthesiology websites. We will use controlled vocabulary and free text terms, with field labels, Boolean, and proximity operators tailored to each search engine. The evaluation period will run from January 2012 to December 2022.

### Data extraction

Since we will use CPGs as the primary source of trials, the data extraction process will consist of two steps.

In the first step, two independent reviewers will screen the search results to identify the CPGs that meet the eligibility criteria. Any disagreements will be resolved through discussion and, if necessary, with the involvement of a third reviewer. Rayyan, a web and mobile app for systematic reviews [24], will be used for the screening process.

In the second step, two independent reviewers will screen the trials identified from each of the included CPGs and data extraction will be conducted using a standardized extraction form in REDCap®.

Since there is the possibility that an RCT is cited in more than one guideline, we will remove duplicates from the data set before the extraction, to avoid taking into account the information of a trial more than once in the analysis.

The extracted data will encompass general information about the CPGs, such as title, year of publication, target audience, total number of recommendations, recommendation classification system used to determine the level of evidence, quality of the studies, and the strength of recommendations. Additionally, we will collect individual study characteristics, publication year, type of trial, unit of allocation, number of participating centers, type of blinding, method of allocation concealment, ethical approval, source of funding, and data sharing agreement. Furthermore, we will extract outcome-related information, outcome name, outcome definition, imputation method, sample size, number of patients randomized per group, number of patients who experienced the endpoint per group, level of statistical significance, and number of patients lost to follow-up.

This study complies with the Preferred Reporting Items for Systematic Reviews and Meta-Analyses (PRISMA) guidelines for methodological studies [25] “S1 Checklist”.

### Sample size estimation

The reference population corresponds to all RCTs identified from the guidelines published between 2012 to 2022, and the unit of analysis is RCT reports.

As it is a methodological survey a sample of the reference population will be calculated using a “rule of thumb” of 10 events per predictor variable (EPP).

The sample size and EPP calculations were implemented in R using the *sampler* package [26].

The proportion of RCTs required by the sampler package to calculate a stratified sample based on the distribution of RCTs by categories (general, cardiovascular, pediatric, regional, obstetric, and neuroanesthesia) will be obtained from the survey results.

### Data analysis

#### Primary analysis

A descriptive analysis will be conducted to characterize the CPGs and RCTs that support the recommendations. Counts and percentages will be used as summary measures for categorical variables. In contrast, means and interquartile ranges will be used for continuous variables, with standard deviations or ranges employed as appropriate. The Statistical Analyses and Methods in the Published Literature (SAMPL) Guidelines for reporting descriptive statistics will be followed [27].

The FI calculation for RCTs with significant results (p < 0.05), will use the smaller number of events required to obtain a p ≥ 0.05 (4). For non-significant RCTs (p ≥ 0.05), the rFI will be the fewer number of events required to obtain p < 0.05 (5). For the analysis, priority will be given to the outcome(s) supporting the guideline recommendation. Median and interquartile ranges will be used to describe the results.

The overall FI report is tentatively proposed for the following subgroups based on the targeted population (general anesthesia, regional, pediatrics, obstetrics, cardiovascular and neuroanesthesia) of the identified RCTs.

R will be used as statistical software [28], and the R Packages: Fragility Index [29] and FragilityTools [30].

#### Secondary analysis

The exploratory analysis of the factors associated with the overall Fragility Index (FI) will be addressed using a Poisson regression analysis. In case of over-dispersion, a negative binomial regression will be employed. The overall FI will serve as the dependent variable in the analysis. The following independent variables are tentatively proposed to be included in the analysis: (1) type of trial, (2) type of blinding, (3) allocation concealment, (4) patients lost-to-follow-up, (5) source of funding, (6) ethical approval, (7) type of intervention (drug-related/non-drug-related), (8) open data/transparency agreement, and (9) type of imputation method used.

Before fitting the model, the assumptions of Poisson distribution and the absence of over-dispersion will be verified. The maximum likelihood estimation method will be utilized for model fitting, and goodness-of-fit and deviance will be used for model evaluation. Additionally, variance inflation factors (VIF) will be calculated to assess multicollinearity, defined as VIF > 10. The analysis results will be reported as estimated β coefficients with 95% confidence intervals (CIs), p-values for each included variable, and overall model fit statistics. All estimates will be reported to two decimal places, and p-values will be reported to three decimal places.

The R statistical software [28] will be utilized for all analyses. Two-sided hypothesis testing will explore associations between factors and fragility index, with a significance level set at alpha = 0.05.

Refer to **Table 1**. for a summary of the analysis plan.

**Table 1:** Planned statistical analysis summary.

Objective	Outcome	Explanatory variables	Hypothesis	Method of analysis
Primary objective	FI	NA	NA	Descriptive statistics reported as an estimate of FI (95% CI) Results will be reported for overall FI, group FI by targeted population of the trials.
Secondary objective				
	FI	• Type of trial • Type of blinding • Allocation concealment • Patients lost-to-follow-up • Source of funding* • Ethical approval* • Type of intervention • Open data/transparency agreement* • Type of imputation method*	Each factor will be associated with FI	Poisson regression (or negative Binomial)

*Will be included in the final model only if these reach statistical significance (p< 0.05) in the model with all the variables included. FI = fragility index

### Updates and amendments

Updates and amendments to this protocol (if applicable) will be summarized in the final manuscript.

### Ethics and dissemination

This study is a methodological survey and does not involve human subjects. Nevertheless, it has institutional board approval (Act N° 6/2023).

## 3. Discussion

Perioperative medicine faces unique challenges in conducting randomized controlled trials (RCTs) and generating statistically significant results [11]. These limitations have led to the proposal of complementary measures, such as fragility assessment, to determine the robustness of RCT results.

Since the Fragility analysis description, there have been efforts to expand its applicability to other types of outcomes and meta-analysis evaluation [5, 31, 32].

Previous FI assessment of RCTs in CPGs have been limited either to the evaluation of studies published in high-impact journals or to evaluations of a single guideline and its respective RCTs [11, 17, 18].

This proposal, unlike previous research on frailty, focuses on conducting a comprehensive and reproducible search. It does not restrict itself to general anesthesia guidelines, specific types of publications (such as Q1 journals), or only trials with statistically significant results. Consequently, the full spectrum of recommendations and their corresponding randomized controlled trials (RCTs) were included in the sampling frame. This approach aligns with established guidelines for conducting methodological research [23].

The results of this study will provide valuable insights into the use of fragility assessment to show the inherent weakness of depending solely on statistical significance and its futility. And emphasizing its ability to support RCT comparability in perioperative medicine.

## Supporting information

S1 Checklist PRISMA-P-checklist. (DOC)

S1 Appendix Preliminary search. (DOCX)
