# Supplementary material for: Assessing the fragility index of randomized controlled trials supporting perioperative care guidelines: A methodological survey protocol
Source: PLoS One. 2024 Sep 12;19(9):e0310092. doi: 10.1371/journal.pone.0310092 (PMC11392262; doi:10.1371/journal.pone.0310092)
Supplement: S1 Appendix — (DOCX) [file pone.0310092.s002.docx]

**S1 Appendix. Preliminary search**

| **EMBASE search (1659 results) Date: 27/04/2023** |
| --- |
| #33 AND (2012:py OR 2013:py OR 2014:py OR 2015:py OR 2016:py OR 2017:py OR 2018:py OR 2019:py OR 2020:py OR 2021:py OR 2022:py) |
| #25 AND #32 |
| #26 OR #27 OR #28 OR #29 OR #30 OR #31 |
| 'evidence based practice':de |
| 'practice guideline':de |
| 'task force':ti,ab |
| 'evidence based':ti,ab |
| 'guideline*':ti,ab |
| 'practice guideline':ti,ab |
| #1 OR #2 OR #3 OR #4 OR #5 OR #6 OR #7 OR #8 OR #9 OR #10 OR #11 OR #12 OR #13 OR #14 OR #15 OR #16 OR #17 OR #18 OR #19 OR #20 OR #21 OR #22 OR #23 OR #24 |
| 'canadian anesthesiologists society':ti,ab |
| 'royal college of anaesthetists':ti,ab |
| 'eras society':ti,ab |
| 'difficult airway society':ti,ab |
| 'obstetric anaesthetists association':ti,ab |
| 'neuroanaesthesia and critical care society':ti,ab |
| 'european association of cardiothoracic anesthesiology':ti,ab |
| 'european society for paediatric anaesthesiology':ti,ab |
| 'european society of regional anaesthesia and pain therapy':ti,ab |
| 'european society of anaesthesiology':ti,ab |
| 'american pain society':ti,ab |
| 'american society for enhanced recovery':ti,ab |
| 'society for the advancement of transplant anesthesia':ti,ab |
| 'society of academic associations of anesthesiology and perioperative medicine':ti,ab |
| 'society for airway management':ti,ab |
| 'society for neuroscience in anesthesiology and critical care':ti,ab |
| 'trauma anesthesiology society':ti,ab |
| 'society for pediatric anesthesia':ti,ab |
| 'society of critical care anesthesiologists':ti,ab |
| 'society of anesthesia and sleep medicine':ti,ab |
| 'society of cardiovascular anesthesiologists':ti,ab |
| 'society for obstetric anesthesia and perinatology':ti,ab |
| 'american society of regional anesthesia and pain medicine':ti,ab |
| 'american society of anesthesiologists':ti,ab |

| **MEDLINE search (956 results) Date: 27/04/2023** |
| --- |
| (((((((((((((((((((((((((("society for obstetric anesthesia and perinatology"[tiab:~0]) OR ("american society of anesthesiologists"[tiab:~0])) OR ("american society of regional anesthesia and pain medicine"[tiab:~0])) OR ("society of cardiovascular anesthesiologists"[tiab:~0])) OR ("society for ambulatory anesthesia"[tiab:~0])) OR ("society of anesthesia and sleep medicine"[tiab:~0])) OR ("society of critical care anesthesiologists"[tiab:~0])) OR ("society for pediatric anesthesia"[tiab:~0])) OR ("trauma anesthesiology society"[tiab:~0])) OR ("society for neuroscience in anesthesiology and critical care"[tiab:~0])) OR ("society for airway management"[tiab:~0])) OR ("society of academic associations of anesthesiology and perioperative medicine"[tiab:~0])) OR ("society for the advancement of transplant anesthesia"[tiab:~0])) OR ("american society for enhanced recovery"[tiab:~0])) OR ("american pain society"[tiab:~0])) OR ("european society of anaesthesiology"[tiab:~0])) OR ("european society of regional anaesthesia and pain therapy"[tiab:~0])) OR ("european society for paediatric anaesthesiology"[tiab:~0])) OR ("european association of cardiothoracic anesthesiology"[tiab:~0])) OR ("neuroanaesthesia and critical care society"[tiab:~0])) OR ("obstetric anaesthetists association"[tiab:~0])) OR ("difficult airway society"[tiab:~0])) OR ("eras society"[tiab:~0])) OR ("association of anaesthetists"[tiab:~0])) OR ("royal college of anaesthetists"[tiab:~0])) OR ("canadian anesthesiologists society"[tiab:~0])) AND (((((("practice guideline"[Title/Abstract]) OR (guideline*[Title/Abstract])) OR (evidence-based[Title/Abstract])) OR ("task force"[Title/Abstract])) OR (practice guideline[MeSH Terms])) OR (evidence based medicine[MeSH Terms])) |
| (((((((((((((((((((((((((("society for obstetric anesthesia and perinatology"[tiab:~0]) OR ("american society of anesthesiologists"[tiab:~0])) OR ("american society of regional anesthesia and pain medicine"[tiab:~0])) OR ("society of cardiovascular anesthesiologists"[tiab:~0])) OR ("society for ambulatory anesthesia"[tiab:~0])) OR ("society of anesthesia and sleep medicine"[tiab:~0])) OR ("society of critical care anesthesiologists"[tiab:~0])) OR ("society for pediatric anesthesia"[tiab:~0])) OR ("trauma anesthesiology society"[tiab:~0])) OR ("society for neuroscience in anesthesiology and critical care"[tiab:~0])) OR ("society for airway management"[tiab:~0])) OR ("society of academic associations of anesthesiology and perioperative medicine"[tiab:~0])) OR ("society for the advancement of transplant anesthesia"[tiab:~0])) OR ("american society for enhanced recovery"[tiab:~0])) OR ("american pain society"[tiab:~0])) OR ("european society of anaesthesiology"[tiab:~0])) OR ("european society of regional anaesthesia and pain therapy"[tiab:~0])) OR ("european society for paediatric anaesthesiology"[tiab:~0])) OR ("european association of cardiothoracic anesthesiology"[tiab:~0])) OR ("neuroanaesthesia and critical care society"[tiab:~0])) OR ("obstetric anaesthetists association"[tiab:~0])) OR ("difficult airway society"[tiab:~0])) OR ("eras society"[tiab:~0])) OR ("association of anaesthetists"[tiab:~0])) OR ("royal college of anaesthetists"[tiab:~0])) OR ("canadian anesthesiologists society"[tiab:~0])) AND (((((("practice guideline"[Title/Abstract]) OR (guideline*[Title/Abstract])) OR (evidence-based[Title/Abstract])) OR ("task force"[Title/Abstract])) OR (practice guideline[MeSH Terms])) OR (evidence based medicine[MeSH Terms])) |
| ((((("practice guideline"[Title/Abstract]) OR (guideline*[Title/Abstract])) OR (evidence-based[Title/Abstract])) OR ("task force"[Title/Abstract])) OR (practice guideline[MeSH Terms])) OR (evidence based medicine[MeSH Terms]) |
| evidence based medicine[MeSH Terms] |
| practice guideline[MeSH Terms] |
| "task force"[Title/Abstract] |
| evidence-based[Title/Abstract] |
| guideline*[Title/Abstract] |
| "practice guideline"[Title/Abstract] |
| ((((((((((((((((((((((((("society for obstetric anesthesia and perinatology"[tiab:~0]) OR ("american society of anesthesiologists"[tiab:~0])) OR ("american society of regional anesthesia and pain medicine"[tiab:~0])) OR ("society of cardiovascular anesthesiologists"[tiab:~0])) OR ("society for ambulatory anesthesia"[tiab:~0])) OR ("society of anesthesia and sleep medicine"[tiab:~0])) OR ("society of critical care anesthesiologists"[tiab:~0])) OR ("society for pediatric anesthesia"[tiab:~0])) OR ("trauma anesthesiology society"[tiab:~0])) OR ("society for neuroscience in anesthesiology and critical care"[tiab:~0])) OR ("society for airway management"[tiab:~0])) OR ("society of academic associations of anesthesiology and perioperative medicine"[tiab:~0])) OR ("society for the advancement of transplant anesthesia"[tiab:~0])) OR ("american society for enhanced recovery"[tiab:~0])) OR ("american pain society"[tiab:~0])) OR ("european society of anaesthesiology"[tiab:~0])) OR ("european society of regional anaesthesia and pain therapy"[tiab:~0])) OR ("european society for paediatric anaesthesiology"[tiab:~0])) OR ("european association of cardiothoracic anesthesiology"[tiab:~0])) OR ("neuroanaesthesia and critical care society"[tiab:~0])) OR ("obstetric anaesthetists association"[tiab:~0])) OR ("difficult airway society"[tiab:~0])) OR ("eras society"[tiab:~0])) OR ("association of anaesthetists"[tiab:~0])) OR ("royal college of anaesthetists"[tiab:~0])) OR ("canadian anesthesiologists society"[tiab:~0]) |
| "canadian anesthesiologists society"[tiab:~0] |
| "royal college of anaesthetists"[tiab:~0] |
| "association of anaesthetists"[tiab:~0] |
| "eras society"[tiab:~0] |
| "difficult airway society"[tiab:~0] |
| "obstetric anaesthetists association"[tiab:~0] |
| "neuroanaesthesia and critical care society"[tiab:~0] |
| "european association of cardiothoracic anesthesiology"[tiab:~0] |
| "european society for paediatric anaesthesiology"[tiab:~0] |
| "european society of regional anaesthesia and pain therapy"[tiab:~0] |
| "european society of anaesthesiology"[tiab:~0] |
| "american pain society"[tiab:~0] |
| "american society for enhanced recovery"[tiab:~0] |
| "society for the advancement of transplant anesthesia"[tiab:~0] |
| "society of academic associations of anesthesiology and perioperative medicine"[tiab:~0] |
| "society for airway management"[tiab:~0] |
| "society for neuroscience in anesthesiology and critical care"[tiab:~0] |
| "trauma anesthesiology society"[tiab:~0] |
| "society for pediatric anesthesia"[tiab:~0] |
| "society of critical care anesthesiologists"[tiab:~0] |
| "society of anesthesia and sleep medicine"[tiab:~0] |
| "society for ambulatory anesthesia"[tiab:~0] |
| "society of cardiovascular anesthesiologists"[tiab:~0] |
| "american society of regional anesthesia and pain medicine"[tiab:~0] |
| "american society of anesthesiologists"[tiab:~0] |
| "society for obstetric anesthesia and perinatology"[tiab:~0] |
